# Acute myeloneuropathy after nitrous oxide use: multimodal diagnostic insights: Response to research article: increase of nitrous oxide-induced neurological disorders – a German multicenter experience by Meißner et al.

**DOI:** 10.1186/s42466-025-00444-6

**Published:** 2025-11-14

**Authors:** Julius Runzheimer, Omar Alhaj Omar, Martin Jünemann, Alexander Brose, Tobias Struffert, Heidrun H. Krämer, Steffen Pfeuffer, Anne Mrochen

**Affiliations:** 1 Department of Neurology, University Hospital Giessen and Marburg, Justus-Liebig- University Giessen, Giessen, Germany; 2 Department of Neuroradiology, University Hospital Giessen and Marburg, Justus- Liebig-University Giessen, Giessen, Germany

We read with great interest the multicenter report by Meißner et al. highlighting the increasing incidence of nitrous oxide (N₂O)-induced neurological disorders [1]. We would like to complement their findings by emphasizing diagnostic considerations, which are crucial for the timely identification of N₂O-related neurotoxicity.

We report the case of a 24-year-old male who developed progressive subacute myeloneuropathy after several months of recreational N₂O use via whipped cream chargers. His initial symptoms—numbness in the soles of the feet—progressed to severe sensory ataxia and paraparesis, ultimately requiring wheelchair use.

A key diagnostic challenge in this case was the misleadingly normal serum vitamin B12 level (157 pg/ml; reference 150–900 pg/ml) and holotranscobalamin (64 pmol/l; reference > 60 pmol/l). However, functional B12 deficiency was clearly demonstrated by significantly elevated methylmalonic acid (13,512 nmol/l; reference 50–450 nmol/l) and homocysteine levels (142 µmol/l; reference < 12µmol/l), underscoring the limited diagnostic utility of total B12 or holotranscobalamin measurements alone in N₂O-related cases. Of note, further analyses including CSF and whole blood count were normal. Especially, megaloblastic anemia was absent.

Spinal MRI revealed T2-hyperintensities in the dorsal columns of the cervical and thoracic spine, forming the characteristic “inverted V-sign,” consistent with subacute myelopathy (Fig. 1) [2, 4]. In addition, nerve conduction studies confirmed a length-dependent sensorimotor neuropathy with severe axonal loss in the lower limbs (Table 1). Notably, serum neurofilament light chain (NfL) levels were markedly elevated (51.3 pg/ml; reference 0–10.7 pg/ml), indicating significant neuroaxonal damage. NfL appears promising for differential diagnosis of acute flaccid weakness since recent data indicated only minor increases in acute demyelinating neuropathy which often appears as differential diagnosis for N_2_O-intoxication [5].

**Fig. 1:**
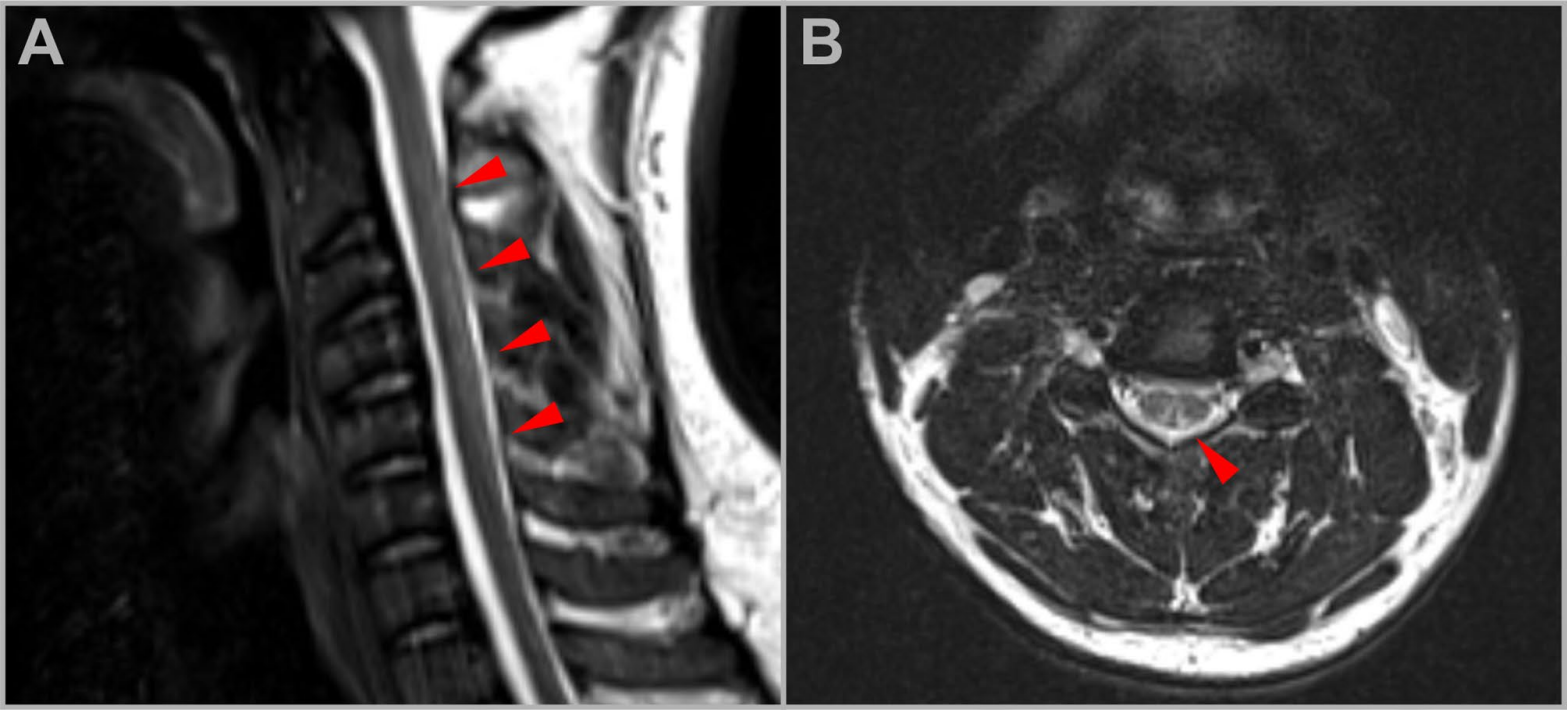
MRI findings in N₂O-associated myelopathy. Sagittal (**A**) and axial (**B**) T2-weighted MRI images of the cervical spine. (**A**) Sagittal T2-weighted image demonstrates longitudinally extensive hyperintense signal abnormalities within the dorsal columns of the cervical spinal cord (red arrowheads). (**B**) Axial T2-weighted image shows a characteristic V-shaped hyperintensity involving the dorsal aspect of the spinal cord (red arrowhead), consistent with selective involvement of the posterior columns

**Table 1:** Nerve conduction studies indicating severe axonal loss within the lower limbs

Motor recording				
Nerve (right/left)	**dmL (ms)**	**CMAP (mV)**	**NCS (m/s)**	**F-wave (ms)**
Median	3.95/4.66 (4.2)	8.2/9.3 (8)	51.1/53.6 (50)	32.8/33.2 (30)
Ulnar	3.03/3.17 (3.5)	13.3/10.7 (8)	51.9/55.0 (50)	31.2/33.1 (30)
Tibial	-/-	-/-	-/-	-/-
Peronal	-/-	-/-	-/-	-/-

**Sensory recording**				
Nerve (right/left)	**SAP (µV)**	**NCS (m/s)**	**N20/P40 (ms)**	**Amp (µV)**
Median	21.8/15.6 (12)	45.8/44.2 (50)	24.1/23.3(22.3)	2.4/2.6
Ulnar	11.2/17.5 (15)	50.9/49.8 (50)		
Tibial			-/-	-/-
Sural	-/-	-/-		

Additionally, we noticed an asymptomatic left-sided carpel tunnel syndrome. Reference ranges are given in brackets. Abbreviations: dmL: distal motor latency; CMAP: compound muscle action potential; NCS: nerve conduction speed; SAP: sensory action potential; N20: N20 latency; P40: P40 latency Amp: amplitude

The patient was diagnosed with N₂O-induced subacute combined myelopathy and axonal neuropathy. High-dose intramuscular hydroxocobalamin (1,000 µg daily for one week, then weekly) was initiated, alongside strict cessation of N₂O. Following cessation, the patient developed significant apathy and depressive symptoms, which required further neuropsychiatric evaluation and support. Despite treatment, paraparesis persisted and the patient was subjected to rehabilitation.

This case reinforces several important diagnostic insights:

1. Normal serum B12 and holotranscobalamin levels do not exclude functional deficiency, particularly in N₂O-induced cases. Elevated methylmalonic acid and homocysteine remain the more sensitive indicators.
2. Spinal MRI with dorsal column hyperintensities and the “inverted V-sign” can serve as a valuable early radiological marker.
3. Elevated serum neurofilament light chain may serve as a useful biomarker of active neurodegeneration and should be further evaluated in future studies.

Given the increasing recreational use of N₂O particularly among young adults, clinicians should consider it as an important differential diagnosis when encountering unexplained myelopathy, sensory ataxia, or neuropathy. Early identification, discontinuation of exposure, and parenteral B12 replacement are essential to prevent irreversible damage. Finally, we echo the call for urgent policy reassessment regarding the unregulated accessibility of nitrous oxide [1].

## Data Availability

The data supporting the findings of this case report are available from the corresponding author upon reasonable request.
